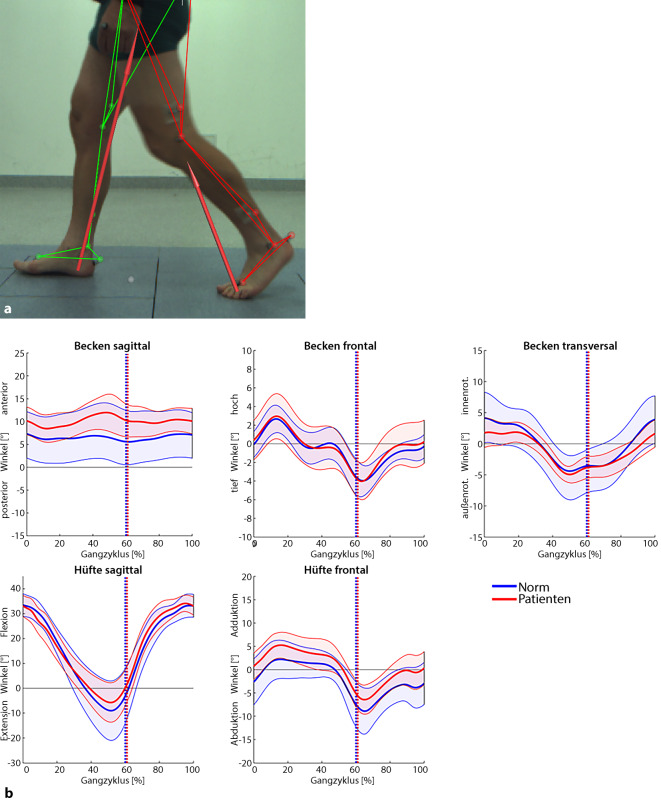# Der Pararectus-Zugang: operatives Vorgehen in der Acetabulumchirurgie

**DOI:** 10.1007/s00064-023-00800-2

**Published:** 2023-03-16

**Authors:** Christian von Rüden, Andreas Brand, Mario Perl

**Affiliations:** 1 Abteilung Unfallchirurgie, BG Unfallklinik Murnau, Murnau, Deutschland; 2 Universitätsinstitut für Biomechanik, Paracelsus Medizinische Privatuniversität, Salzburg, Österreich; 3 Unfallchirurgische und Orthopädische Klinik, Universitätsklinikum Erlangen, Friedrich-Alexander Universität Erlangen-Nürnberg (FAU), Krankenhausstr. 12, 91054 Erlangen, Deutschland

**Keywords:** Acetabulumfraktur, Quadrilaterale Fläche, Interne Osteosynthese, Vorderer Pfeiler/vordere Wand Fraktur, Ganganalyse, Acetabular fracture, Internal fracture fixation, Quadrilateral surface, Anterior column/anterior wall fracture, Gait analysis

## Abstract

**Operationsziel:**

Der Pararectus-Zugang wurde vor einigen Jahren für die Beckenchirurgie neu entdeckt und als alternativer Zugang insbesondere für die Behandlung von Acetabulumfrakturen des vorderen Pfeilers mit Beteiligung der quadrilateralen Fläche beschrieben.

**Indikationen:**

Zur optimalen Darstellung von Acetabulumfrakturen mit Beteiligung der quadrilateralen Fläche, Frakturen der vorderen Wand und des vorderen Pfeilers, vorderen Pfeiler/hinteren Hemiquerfrakturen und Frakturen mit zentraler Impression von Domfragmenten hat sich der Pararectus-Zugang bewährt.

**Kontraindikationen:**

Bei Frakturen des hinteren Pfeilers, der hinteren Wand, kombinierten Frakturen der hinteren Wand und des hinteren Pfeilers, Querfrakturen mit Dislokation des hinteren Pfeilers oder in Kombination mit Frakturen der hinteren Wand und bei T‑Frakturen mit Dislokation des hinteren Pfeilers oder in Kombination mit Frakturen der hinteren Wand findet der Pararectus-Zugang keine Anwendung, ebenso nicht bei Patienten mit Ileus.

**Operationstechnik:**

Über den Pararectus-Zugang kann der gesamte Beckenring einschließlich der quadrilateralen Fläche erreicht werden. Die Auswahl der chirurgischen Fenster richtet sich nach der Frakturlokalisation und den Erfordernissen der Frakturreposition.

**Weiterbehandlung:**

Grundsätzlich sollte eine Teilbelastung für 6 Wochen eingehalten werden, wobei Fraktur- und Osteosynthese-abhängig ggf. eine frühere Belastungsfreigabe möglich ist. Insbesondere von geriatrischen Patienten kann häufig keine Teilbelastung eingehalten werden, sodass hier die frühzeitige und oft relativ unkontrollierte Vollbelastung akzeptiert werden muss.

**Ergebnisse:**

In einer vergleichenden instrumentellen Bewegungsanalyse zwischen Patienten nach operativer Stabilisierung einer isolierten unilateralen Acetabulumfraktur über den Pararectus-Zugang und gesunden Probanden zeichnete sich schon in der frühen postoperativen Phase eine suffiziente Stabilität und Bewegungsfunktion des Beckens und der Hüfte während des Gehens ab.

## Abstract (en)

**Objective:**

The pararectus approach was rediscovered several years ago for pelvic surgery and described as an alternative approach especially for the treatment of acetabular fractures of the anterior column involving the quadrilateral plate.

**Indications:**

For optimal visualization of acetabular fractures involving the quadrilateral plate, fractures of the anterior wall and anterior column, anterior column/posterior hemitransverse fractures, and fractures with central impression of dome fragments, the pararectus approach has proven to be a useful access.

**Contraindications:**

The pararectus approach is not used for posterior column fractures, posterior wall fractures, combined posterior wall and posterior column fractures, transverse fractures with displaced posterior column or in combination with posterior wall fractures, and T‑fractures with displaced posterior column or in combination with posterior wall fractures.

**Surgical technique:**

The entire pelvic ring, including the quadrilateral plate, can be accessed via the pararectus approach. The choice of the correct surgical window depends on the fracture location and the requirements of fracture reduction.

**Postoperative management:**

In general, partial weight-bearing should be maintained for 6 weeks, although earlier weight-bearing release may be possible if necessary, depending on fracture pattern and osteosynthesis. Particularly in geriatric patients, partial weight-bearing is often not possible, so that early and often relatively uncontrolled full weight-bearing has to be accepted.

**Results:**

In a comparative gait analysis between patients following surgical stabilization of an isolated unilateral acetabular fracture through the pararectus approach and healthy subjects, sufficient stability and motion function of the pelvis and hip during walking was already evident in the early postoperative phase.

## Vorbemerkungen

Der Pararectus-Zugang, der früher in der Urologie und in der Wirbelsäulenchirurgie zur Darstellung des L5/S1-Segments verwendet wurde, wurde vor einigen Jahren für die Beckenchirurgie neu entdeckt und als alternativer Zugang zum ilioinguinalen und zum modifizierten Stoppa-Zugang insbesondere für die Behandlung von Acetabulumfrakturen mit Beteiligung der quadrilateralen Fläche beschrieben [1–7]. Er kann sinnvollerweise mit dem ersten Fenster des ilioinguinalen Zugangs kombiniert werden, um so im Rendezvous-Verfahren vollständigen Zugang zur inneren Darmbeinschaufel bis hinauf zur Crista iliaca zu erhalten. Außerdem erleichtert er die optionale Insertion einer supra- und/oder infraazetabulären Schraube über den korrekten Korridor oder andere hilfreiche Repositionsmanöver wie die Anwendung einer auxiliären Cerclage [8, 9]. Darüber hinaus ist der Pararectus-Zugang bei Patienten mit vorbestehender Leistenhernie oder bei Patienten mit Peritonealnetz sinnvoll einsetzbar. In diesem Fall muss das Netz im Gegensatz zum ilioinguinalen Zugang – wenn überhaupt – nur über eine sehr kurze Distanz inzidiert werden, ohne dass ausgedehnte Narben gelöst werden müssen [10, 11]. Außerdem hat sich der Pararectus-Zugang für die Revisionssituation nach primär angewendetem ilioinguinalen Zugang bewährt. Heute wird der Stoppa-Zugang in modifizierter Form mit Mittellinieninzision zunehmend als vorderer Standardzugang angesehen [12–14], der bei Polytraumatisierung auch die Möglichkeit bietet, intraabdominelle Verletzungen bilateral zu erreichen [15]. Auch wenn man kosmetische Überlegungen mit einbezieht, bietet der Stoppa-Zugang, wenn er als Pfannenstielinzision angelegt ist, Vorteile [16].

## Operationsprinzip und -ziel

- Wenig invasive Stabilisierung von Acetabulumfrakturen insbesondere der vorderen Säule und vorderen Wand, mit Dislokation der quadrilateralen Fläche und mit Impaktion des Pfannendachs über den Pararectus-Zugang
- Darstellung der externen Iliakalgefäße, der unteren Bauchwandgefäße, der Obturatorgefäße und der Corona mortis, bis die Fraktur gut eingesehen werden kann
- Reposition der Fragmente unter Sicht und Stabilisierung mit anatomisch präkonturierter Kleinfragmentplatte und ggf. Einzelschrauben

Ziel ist die möglichst stufenfreie anatomische Reposition und Stabilisierung der Fraktur zur Vermeidung einer sekundären Arthrose und schmerzfreien Mobilisation des Patienten.

## Vorteile

- Kombiniert die Vorteile des ilioinguinalen Zugangs mit denen des modifizierten Stoppa-Zugangs insbesondere zu der optimalen Versorgung von Acetabulumfrakturen mit Beteiligung der quadrilateralen Fläche [17–19]
- Möglichkeit einer Reposition, die der Richtung der Frakturdislokation direkt entgegenwirkt
- Möglichkeit, den nach zentral gerichteten Frakturkräften unter direkter Sicht durch den zwischen Peritoneum und lateraler Bauchmuskulatur entwickelten Zugang entgegenzuwirken, was die Reposition deutlich erleichtert
- Verbesserte Repositionsqualität mit mehr als 90 % stufenfreien Repositionsergebnissen [19]
- Ausgeprägte anatomische Vorteile, die zu einem geringeren Zugangstrauma und einer sehr guten anatomischen Freilegung der Fraktur führen
- Limitierte und ästhetisch ansprechende Inzision mit einer durchschnittlichen Länge von nur etwa 10 cm
- Klare anatomische Ebene und gute Übersicht und Kontrolle der wichtigen Blutgefäße und Nerven auf direktem Operationsweg
- Geringe intraoperative Expositionszeit und einfache Progression zum Geweberaum neben dem M. rectus abdominis und dem Retroperitoneum [3]
- In der Regel nur etwa 10 Minuten, um das Peritoneum zu erreichen und den Beckenring freizulegen
- Reposition und Fixierung von Frakturen des medialen Darmbeins, des Beckenrings und der quadrilateralen Fläche unter direkter Sicht durch verschiedene Operationsfenster [2]
- Sehr gute direkte mediale Übersicht insbesondere auf zentral gelegene Frakturfragmente
- Wenig iatrogene Verletzungen der längs verlaufenden Gefäße und Nerven [1]
- Zügige postoperative Genesung und geringes Auftreten von Leistenhernien [20].

## Nachteile

- Potenzielle Verletzung des Peritoneums infolge der Zugangslokalisation zwischen Peritoneum und seitlichen Bauchmuskeln

## Indikationen

- Frakturen mit Beteiligung der quadrilateralen Fläche
- Frakturen der vorderen Wand
- Frakturen des vorderen Pfeilers
- Vordere Pfeiler/hintere Hemiquerfrakturen
- Frakturen mit zentraler Impression von Domfragmenten

## Kontraindikationen

- Fraktur des hinteren Pfeilers
- Fraktur der hinteren Wand
- Kombinierte Fraktur der hinteren Wand und des hinteren Pfeilers
- Querfraktur mit Dislokation vorwiegend des hinteren Pfeilers oder kombiniert mit Fraktur der hinteren Wand
- T‑Fraktur mit Dislokation vorwiegend im Bereich des hinteren Pfeilers oder kombiniert mit Fraktur der hinteren Wand

## Patientenaufklärung

*Zu den allgemeinen Risiken gehören:*

- Infektion
- Verletzung von Muskeln, Nerven und Gefäßen
- Intraoperative Fraktur
- Gelenkluxation
- Sekundäre Dislokation bzw. Repositionsverlust
- Osteosynthesematerialversagen
- Verzögerte Frakturheilung
- Pseudarthrose
- Subfasziales Hämatom insbesondere bei Frakturmustern mit schwerer Dislokation
- Venöse Thrombembolie
- Kardiale und/oder pulmonale Komplikationen
- Unklares postoperatives Schmerzsyndrom

*Zu den spezifischen Risiken gehören:*

- Affektion des Nervus (N.) obturatorius
- Affektion des N. cutaneus femoris lateralis
- Affektion der Arteria (A.) und Vena (V.) iliaca, N. femoralis
- Kompressionsbedingte Thrombose der V. iliaca

## Operationsvorbereitung

- Frakturklassifikation
- Wahl und Lage der Osteosynthesematerialien
- Computertomographie inklusive dreidimensionalen (3-D) Rekonstruktionen
- Markierung des Operationssitus durch den Operateur
- Breitbandantibiotikumgabe einmalig etwa 30 Minuten vor Operationsbeginn, Wiederholung nach 3 Stunden Operationszeit
- Steriles Anzeichnen der Landmarken und der geplanten Schnittführung
- Team-Time-out

## Instrumentarium

**Figure 1:**
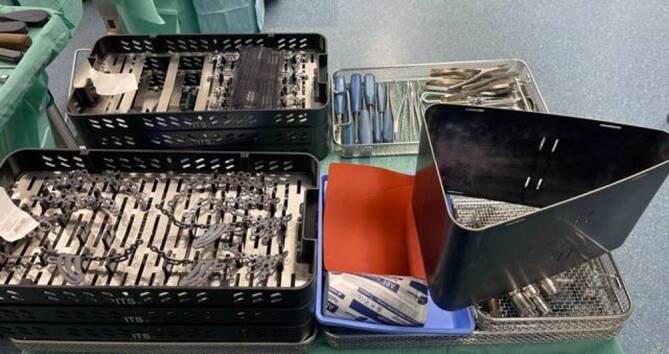

- Routineinstrumente und -implantate für die Osteosynthese von Acetabulumfrakturen in den entsprechenden Zentren standardmäßig vorrätig (Abb. 1)
- Verwendung eines röntgendurchlässigen Karbontisches erleichtert die primäre Implantation einer Hüftgelenkendoprothese über einen minimal-invasiven direkten anterioren Zugang, wenn bei älteren Patienten bereits initial erkannt wird, dass eine Hüftpfannenfraktur nicht mehr anatomisch rekonstruierbar ist
- Statt Seitenstützen Verwendung einer röntgendurchlässigen Vakuummatratze empfehlenswert
- Spezielle röntgendurchlässige Wundhaken in langer Ausführung optional erhältlich
- LED-Einweglampen zur besseren Ausleuchtung des Operationssitus als Aufsatz für die Wundhaken der neuesten Generation optional verfügbar

## Anästhesie und Lagerung

**Figure 2:**
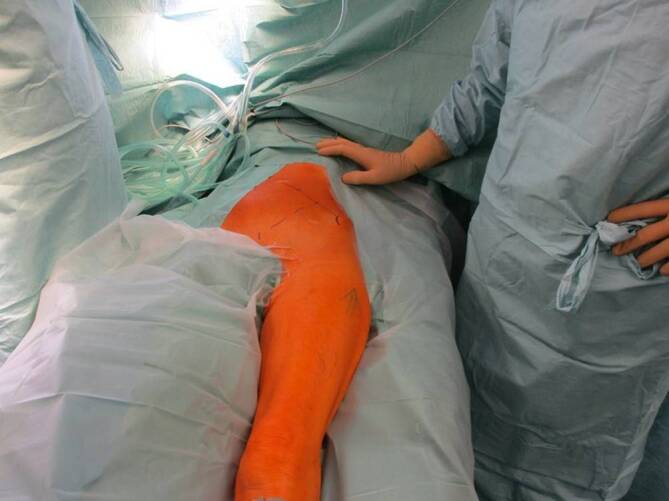

- Vollnarkose für die Osteosynthese von Acetabulumfrakturen erforderlich
- Lagerung des Patienten in Rückenlage auf dem Karbontisch
- Vorbereitung eines Beckensiebs mit Standard- und Spezialinstrumentarium, speziellen Platten und Schrauben der neuesten Generation und Lagerungsdreieck zur Neutralisierung des Musculus (M.) iliopsoas und zur intraoperativen Erleichterung der Frakturreposition und -retention über Beugung im Kniegelenk (Abb. 1)
- Sterile Abdeckung des präoperativ markierten und frei gelagerten verletzten Beins, um intraoperativ Traktion am Bein oder ggf. eine zusätzlich erforderliche Schanz-Schraube über einen T‑Handgriff eindrehen zu können (Abb. 2)
- Relaxation des M. iliopsoas zur zeitweisen intraoperativen Neutralisierung der Hüftbeugekraft insbesondere während des Frakturrepositionsmanövers
- Operationsteam: Operateur und 2 Assistenten
- Position des Operateurs auf der verletzten Seite und der beiden Assistenten auf der Gegenseite
- Platzierung des Bildverstärkers auf der unverletzten Seite
- Ausreichende Muskel(nach)relaxation für das Repositionsmanöver dringend erforderlich

## Operationstechnik

**Figure 3:**
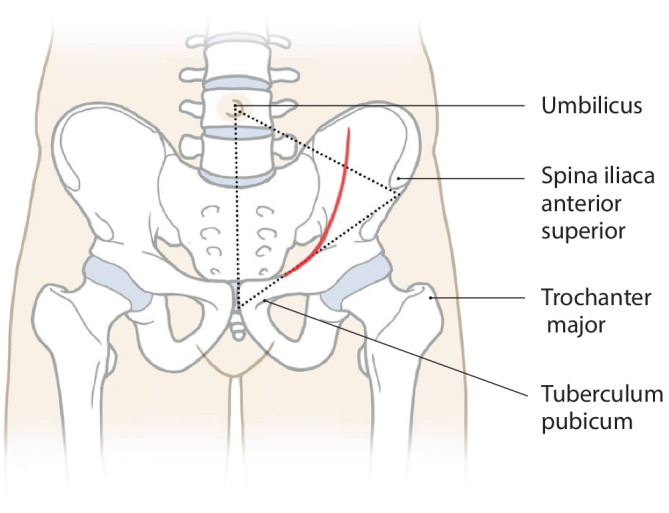

**Figure 4:**
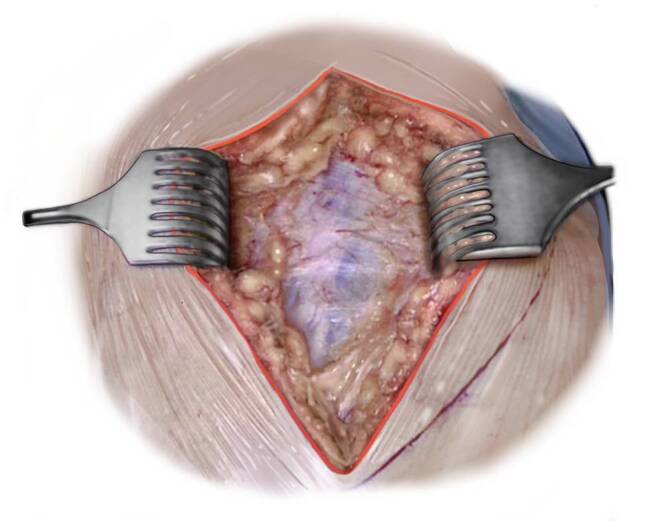

**Figure 5:**
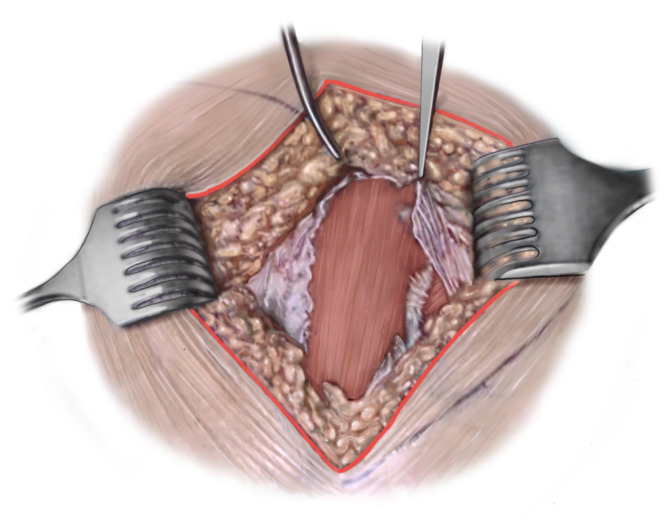

**Figure 6:**
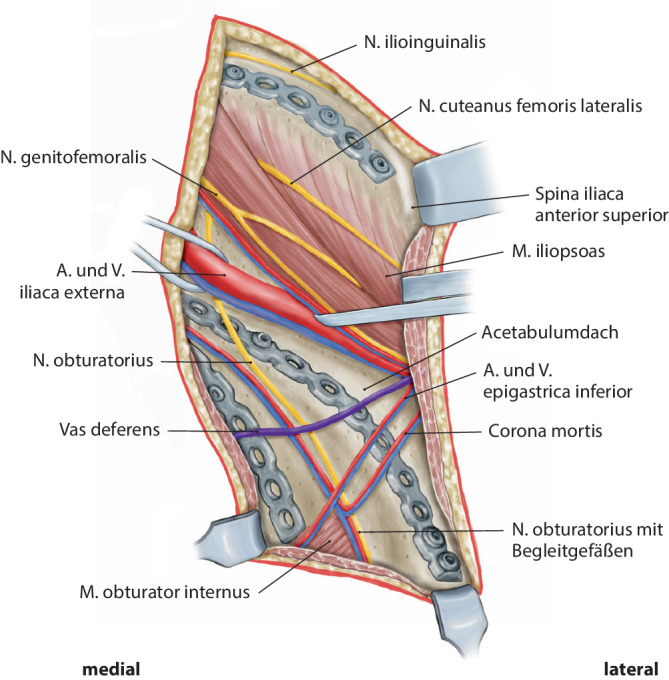

**Figure 7:**
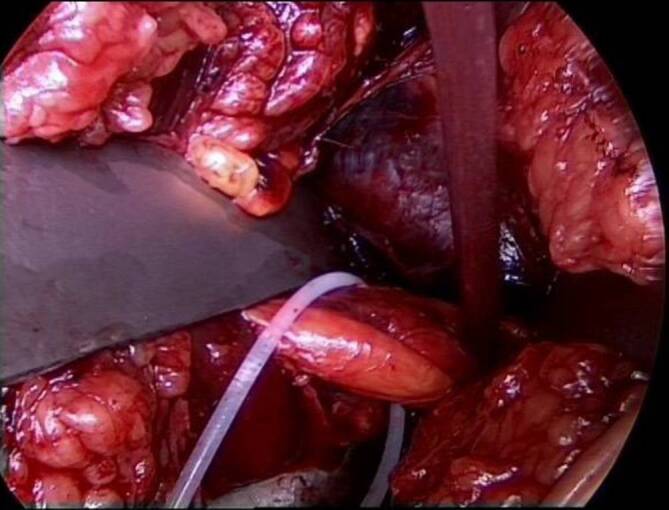

**Figure 8:**
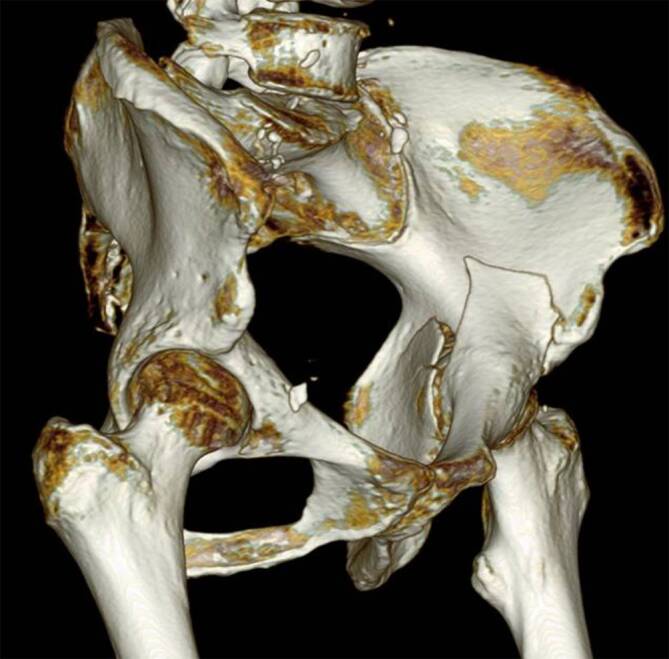

(Abb. 3, 4, 5, 6, 7, 8, 9 und 10)

**Figure 9:**
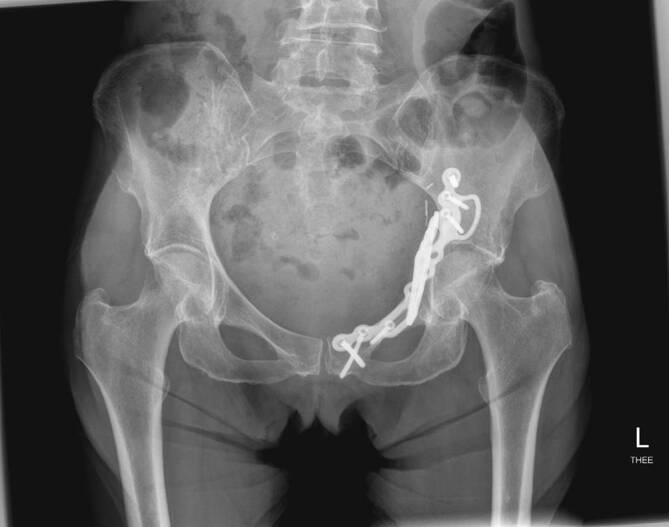

**Figure 10:**
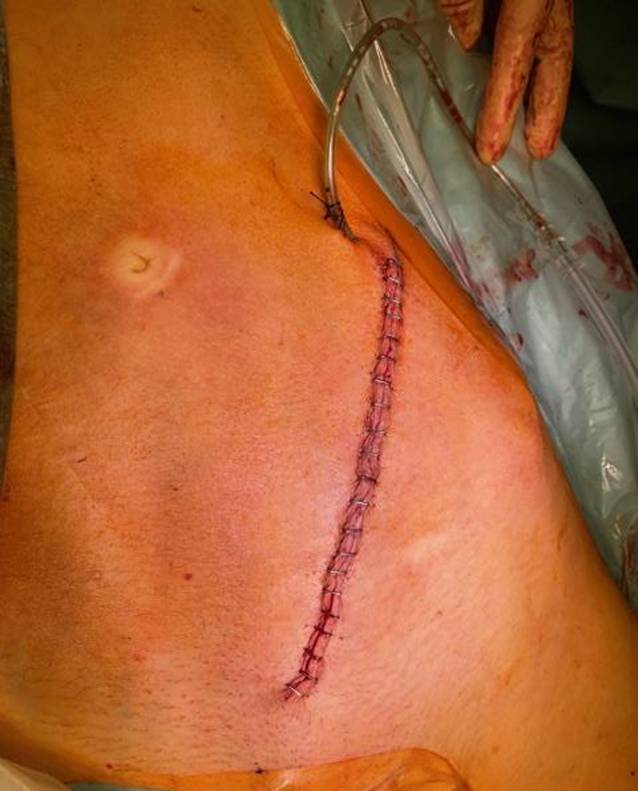

## Postoperative Behandlung

**Figure 11:**
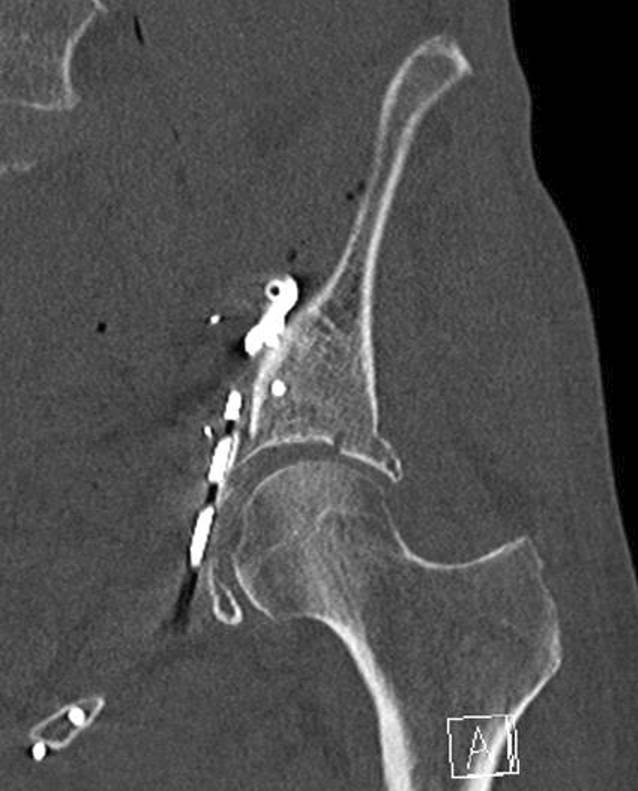

Der Drainageschlauch wird nach der Operation entfernt, sobald das tägliche Drainagevolumen weniger als 50 ml beträgt. Nach dem Drainagezug wird eine CT-Kontrolle des Repositionsergebnisses (Abb. 11) und der Osteosynthesemateriallage inklusive 3‑D-Rekonstruktionen durchgeführt. Routinemäßige konventionelle Röntgenkontrollen mit Ala- und Obturatoraufnahme werden bei normalem Heilverlauf nach 2 und 6 Wochen sowie nach 3, 6 und 12 Monaten empfohlen. Die Nachbehandlung nach operativer Therapie einer Acetabulumfraktur über den Pararectus-Zugang beinhaltet neben der Thromboseprophylaxe die Schmerzreduktion, die Innervationsschulung der Muskulatur und die Reduktion des Lymphödems. Daneben sollen die Geh- und Stehfähigkeit, Selbsthilfefähigkeit und körperliche Belastbarkeit verbessert werden. Die Zielsetzung der Operation sollte präoperativ geklärt werden. Die postoperative Behandlung folgt der präoperativ festgelegten Zielsetzung (anatomische Rekonstruktion vs. TEP vorbereitend; [21]).

Grundsätzlich wird in der rekonstruktiven Acetabulumchirurgie die frühfunktionelle Nachbehandlung angestrebt [22]. Insbesondere im geriatrischen Patientenkollektiv ist es vordringliches Ziel, die frühe postoperative Mobilisation zu ermöglichen. Die betroffene Extremität wird dabei direkt postoperativ unter physiotherapeutischer Supervision mobilisiert, wobei Extrembewegungen vermieden werden sollten. Vor allem bei Frakturen des dorsalen Pfannenrandes ist eine Limitierung der Hüftgelenkbeugung auf 60° sinnvoll. Nach abgeschlossener Wundheilung und erfolgtem Fadenzug kann die frühzeitige Mobilisation über das Wasser begonnen werden. Grundsätzlich sollte eine Teilbelastung von 15–20 kg für 6 Wochen eingehalten werden, wobei Frakturtyp- und Osteosynthese-abhängig ggf. eine frühere Belastungsfreigabe möglich ist. Gerade bei geriatrischen Patienten kann eine Teilbelastung oft nicht umgesetzt werden, sodass die frühzeitige und relativ unkontrollierte Vollbelastung unter Inkaufnahme eines eventuellen Nachsinterns der Fraktur akzeptiert werden muss. Entsprechend der Knochenqualität muss hier mit einer höheren Arthroserate und resultierender sekundärer Prothesenimplantationsrate gerechnet werden [23, 24]. Die Mobilisation bei einseitigen Acetabulumfrakturen erfolgt an 2 Unterarmgehstützen oder initial über einen Unterarmgehwagen, bei beidseitigen Acetabulumfrakturen ist in der Regel primär die Verwendung eines Rollstuhls erforderlich. Auch hier muss ggf. die Hüftgelenkflexion anfangs limitiert werden.

Patienten, die eine Operation des Acetabulums über den Pararectus-Zugang erhalten, sollten regelhaft eine medikamentöse Thromboseprophylaxe zur Vermeidung einer tiefen Beinvenenthrombose erhalten. Grundsätzlich richtet sich die Thromboseprophylaxe nach den aktuell geltenden Empfehlungen der gültigen S3-Leitlinie zur Prophylaxe der venösen Thromboembolie [25]. Bei Patienten unter oraler Antikoagulation ist das perioperative Management ebenfalls weitgehend unproblematisch, unabhängig davon, ob Vitamin-K-Antagonisten oder direkte orale Antikoagulanzien eingesetzt werden [26]. Entscheidend ist ein klinisch vertretbarer Zeitabstand zwischen Unfallereignis und operativem Eingriff. Wird zeitnah zum Unfallereignis operiert, ist mit einem erhöhten Blutungsrisiko zu rechnen. Eine zeitliche Latenz von einigen Tagen kann hier hilfreich sein. Erfolgt der Eingriff dagegen zu verspätet, steigt das Thromboserisiko. Entsprechend muss stets das Blutungs- gegenüber dem Thromboserisiko abgewogen werden.

## Fehler, Gefahren, Komplikationen und ihre Behandlung

- Protrusion des Hüftkopfs nicht korrigierbar → Schanz-Schraube in den Schenkelhals und Reposition
- Operationsgebiet nicht gut einsehbar → maximale Nachrelaxation. Trotz guter Werte im Relaxometer ist der M. psoas oft erst durch eine Nachrelaxation ausreichend mobilisierbar
- Operationsgebiet nicht gut einsehbar → Flexion des Beines und Unterpolsterung, z. B. mittels Lagerungsdreiecks zur Entspannung des M. psoas
- Thrombose postoperativ → leitliniengerechtes Vorgehen
- Blutung/Gefäßläsion intraoperativ → Abstopfen mit Bauchtüchern und Blutungskontrolle, ggf. mit Clips
- Sekundäre Dislokation – sorgfältige Analyse mit CT und ggf. Revision, bei Beteiligung des hinteren Pfeilers Revision von dorsal erwägen
- Während des Zuganges Eröffnung des Peritoneums → Naht des Peritoneums
- Dehnung des N. obturatorius während der Freilegung der quadrilateralen Fläche → Dehnung vermeiden, Vorsicht bei Platzierung des Hohmann Hakens nach medial. Bei akzidentieller Dehnung am Ende der Operation Integrität des Nerven überprüfen, im Operationsbericht vermerken, postoperativ mit dem Patienten besprechen, dokumentieren und abwarten
- Traktionsverletzungen des N. cutaneus femoralis lateralis und/oder des N. femoralis → erhaltene Integrität im Operationsbericht dokumentieren, mit dem Patienten besprechen, zuwarten, ggf. neurologisches Konsil zur Verlaufskontrolle
- Intraoperative Einschätzung des Grades der Wiederherstellung des Hüftgelenkes unsicher → Neben Ala- und Obturatoraufnahmen ist die Durchführung eines intraoperativen CT-Scans oder einer sonstigen 3‑D-Bildgebung hilfreich
- Schraubenpositionierung so wählen, dass bei sekundärer Koxarthrose eine Hüft-TEP problemlos und ohne Schraubenbehinderung platziert werden kann
- Intraoperative Reposition bei fortgeschrittener Osteoporose erschwert → zuerst Auflegen der Platte und Reposition des vorderen gegen den hinteren Pfeiler mit Verwendung einer kollinearen Repositionszange

## Ergebnisse

Eigene klinische und radiologische Nachuntersuchungen ergaben ausgezeichnete funktionelle mittelfristige Ergebnisse im modifizierten Merle d’Aubigné Score, LEFS, WOMAC und SF-36 und zeigten, dass die Reposition von Acetabulumfrakturen über den Pararectus-Zugang altersunabhängig mindestens vergleichbar ist mit dem ilioinguinalen Zugang [3, 27]. Auch hinsichtlich möglicher Komplikationen bestätigten die eigenen Beobachtungen die verfügbare Literatur insofern, dass für den Pararectus-Zugang keine höheren Komplikationsraten als für den Stoppa- und den ilioinguinalen Zugang gefunden wurden [6, 28]. Ein relevanter Vorteil des Pararectus-Zugangs wurde in einer signifikant kürzeren Operationszeit gesehen [19]. In einer Ganganalyse wurden zwar einige biomechanische Einschränkungen entdeckt, die mit verbleibenden Defiziten in der Belastbarkeit und Kraft der Hüftmuskulatur zusammenhängen könnten. Allerdings wurde eine sofortige Wiederherstellung der Mobilität erreicht, indem durch die frühzeitige Operation die Bewegung der unteren Extremitäten und des Beckens erhalten werden konnte [29].

Zur genaueren Beurteilung der biomechanischen Gangfunktion wurden im Rahmen einer aktuellen Analyse anhand einer instrumentellen Bewegungsanalyse 8 männliche Patienten (Alter: 48 ± 14 Jahre, Größe: 183 ± 4 cm, Gewicht: 86 ± 12 kg) mit isolierter unilateraler Acetabulumfraktur mit 8 gesunden Probanden (Alter: 49 ± 13 Jahre, Größe: 181 ± 3,9 cm, Gewicht: 84 ± 10 kg) verglichen. Die Untersuchung der Patienten wurde 3,8 ± 1,3 Monate nach operativer Stabilisierung über den Pararectus-Zugang durchgeführt. Abgesehen von einer erhöhten anterioren Kippung des Beckens in der Sagittalebene und einer leicht erhöhten Hüftadduktion zeigten sich vergleichbare Bewegungsexkursionen des Beckens und Hüftgelenks in den übrigen Bewegungsebenen während der Stand- und Schwungphase (Abb. 12). Dadurch zeichnete sich bereits in der frühen postoperativen Phase nach operativer Versorgung über den Pararectus-Zugang aus funktionell-biomechanischer Sicht eine gute Stabilität und Bewegungsfunktion des Beckens und der Hüfte während des Gehens ab.

**Figure 12:**